# Potential therapeutic benefit of ursodeoxycholic acid in the management of non hepato-biliary upper gastrointestinal disorders

**DOI:** 10.15537/smj.2023.44.5.20220886

**Published:** 2023-05

**Authors:** Yasir M. Khayyat

**Affiliations:** *From the Department of Medicine, Faculty of Medicine, Umm AlQura University, Makkah, Kingdom of Saudi Arabia*.

**Keywords:** ursodeoxycholic, biliary, esophagus, dyspepsia, polyp, Barret’s esophagus

## Abstract

**Objectives:**

To examine the potential therapeutic effects of ursodeoxycholic acid (UDCA) on diseases of the esophagus, stomach, and duodenum.

**Methods:**

A search was conducted using EBSCO, Medline, PubMed, Google Scholar and Web of Science as well as international guidelines using MESH terms for treatment of UDCA for diseases of the upper gastrointestinal disorders in adult humans without regard to publication language or date restrictions.

**Results:**

A total of 256 articles and 22 guidelines were initially identified, and 221 were excluded. Final revision of 13 articles and 22 guidelines confirmed that UDCA is found to have a cytoprotective role in Barret’s esophagus within esophageal disorders, improves abdominal pain in functional dyspepsia, and does not alter Helicobacter pylori colonization or inflammation. Conflicting results are noted regarding the role of UDCA in the duodenum as chemopreventive treatment for familial adenomatous polyposis, with polyps regressing and their growth characteristics improving with low doses (10–25 mg/kg/day). On the contrary, no positive effect was noted upon the combination with Celecoxib and with doses of 1000–2000 mg or 20–30 mg/kg/d. Gastrointestinal side effects were predominantly reported. No side effects necessitated hospitalization or ICU admission.

**Conclusion:**

Ursodeoxycholic acid has a limited therapeutic role in functional dyspepsia. There is promising evidence that it may serve as a chemopreventive for Familial adenomatous polyposis and Barret’s esophagus, although further research is needed to confirm these findings.

**PROSPERO No.: CRD 42021267689**

**B**enign upper gastrointestinal (UGI) pathologies span a wide variety of etiologies consisting of neuromuscular disorders that impair the capability to perform deglutition and swallowing. Acid peptic disorders and mucosal dysplastic changes are other etiologies that predominates as well. Several pharmacotherapies are employed to manage them, with the advent of first generation of proton pump inhibitors (PPI’s) in 1977, a remarkable improvement in the outcomes of acid peptic disorders commenced and several drug prototypes emerged.^1^ However, unmet needs emerged due to poor responses among categories of patients treated with appropriate doses of PPI, which led to an investigation of the reasons. Among them, are functional disorders that may coexist, patient compliance and duodenogastric secretions reflux into the gastric and esophageal lumen.^2-4^ Several early studies have shown that bile secretions are of increasing concentrations in patients with gastroesophageal reflux disease (GERD), as determined by gastric potential of hydrogen (pH), fasting bile acid concentrations, and gastric bilirubin, as the disease progresses from uncomplicated reflux to complicated Barret’s esophagus.^5-8^ The latter mechanism exerts a potent effect on the esophageal mucosa to induce damage to the histological structure, impairment of visceral pain sensation and impairment of esophageal muscular contractions amplitude.^9,10^ Additionally, bile reflux exerts variable degrees of histological gastritis and achlorhydria corresponding to the degree of biliary inflammatory effect on the stomach.^11^ Individuals on high fat diet exhibit peak levels of conjugated bile similar to those measured in the colon after consuming a high fat meal.^12^ Dysplastic changes in the gastrointestinal (GI) tract are caused by chronic exposure to environmental and host factors, particularly chronic acid environments and bile acids. There is evidence that bile acids promote carcinogenesis via several mechanisms, including directdeoxyribonucleic acid (DNA) damage, reduction of apoptosis, oxidative stress, and reactive oxygen species.^12,13^ By utilizing chromoendoscopy and image enhanced endoscopy, strategies have been developed for early detection and prevention of dysplastic changes. However, the use of chemopreventive agents in early stages of dysplasia has been questioned, with agents such as acetyl acetic acid (ASA), non-steroidal anti-inflammatory drugs being recommended. Ursodeoxycholic acid (UDCA) was similarly found to offer anti-inflammatory effect and therefore may halt the ongoing dysplastic process. This review aims to examine the clinical evidence regarding the role of UDCA as a potential therapeutic agent for managing UGI disorders and as a chemopreventive agent in premalignant conditions.

## Methods

A systemic review performed for search terms at basic science and clinical literature within the following major search engines: PubMed, Medline, EMBASE, Google scholar and web of science performed for human and experimental human cell lines or cultures. Additionally, the major gastroenterological societies’ guidelines were reviewed in order to determine their recommendations regarding the use of UDCA in UGI diseases. The search involved the following terms: ursodeoxycholic acid, esophageal, gastric, and duodenal. Details of the search terms and search strategy performed is shown on (Appendix 1). Furthermore, cross references of the listed citations were performed to include more studies. Inclusion criteria were adults, human, no language restriction, and no date restriction. Exclusion criteria were pediatric age, non-English literature, animal or veterinary literature, and literature that discussed hepatic or biliary UGI diseases. Metanalysis is contemplated considering the availability of significant outcome results from the search study results. Quality assessment of the searched articles was performed using Consolidated Standards of Reporting Trials (CONSORT) and STROBE methods.^14,15^ The review is registered at PROSPERO, International Prospective Register of Systemic review, University of York, York UK, 2021.

## Results

**Figure 1:**
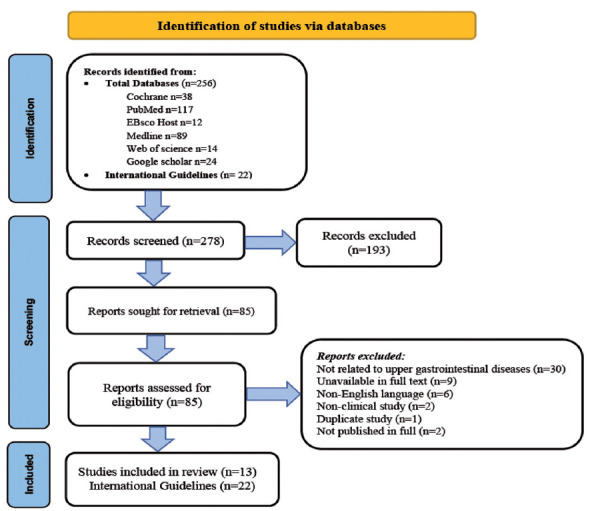
- Search protocol of the role of ursodeoxycholic acid in the management of non hepatobiliary upper gastrointestinal disorders.

A total of 256 articles and 12 international guidelines were initially identified; and 193 articles were excluded upon initial review. Eighty-five articles were sought for further retrieval and review, from which 50 were excluded for the following reasons: articles that were related to upper GI diseases (n=30), studies that were unavailable in full text (n=9), articles not in English (n=6), non-clinical studies (n=2), duplicate studies (n=1), and studies not published in full (n=2) (Figure 1).

In total, 13 articles and 12 international guidelines were included in the systemic review. Quality assessment of the included studies were performed using CONSORT 14 and STROBE 15 methods, and presented in Supplement 1 for the STROBE checklist regarding the randomized trials and Supplement 2 for the CONSORT checklist regarding the observational studies. In reviewing these international guidelines within major gastroenterological societies and associations, it has been found that there are no documented recommendations recommending the use of UDCA in the management of UGI disorders that are endorsed by these societies (Appendix 2).^16-37^ Statistical analysis and useful metanalysis could not be performed due to the small and heterogeneous number of patients present in the clinical studies that were included in the systemic review.

### A. Esophageal disorders

The search revealed several studies on the effect of bile acids on Barret’s esophagus and its dysplastic changes. There are no research or clinical recommendations that show how UDCA affects the spectrum of GERD management, esophageal motility disorders and eosinophilic esophagitis.

**A1. Barret’s esophagus (BE)**. Hydrophobic bile acid deoxycholic acid (DCA) exerts deleterious effect on DNA of Barret’s cells and activation of NF-κβ subunit p65 and its transcriptional activity upon esophageal perfusion in patients who were pretreated with PPI. Peng et al^38^ carried out a randomized study using UDCA. Ursodeoxycholic acid was found to prevent DNA damage in 21 patients who were maintained on omeprazole 20 mg PO (by mouth) twice daily throughout the study^38^ and to significantly increase the messengerRNA (mRNA) and protein expression of the antioxidants studied, namely glutathione peroxidase 1 (GPX1) and catalase, but not superoxide dismutases (SOD1 or SOD2). Furthermore, Pang et al^38^ concluded that 8 weeks of UDCA treatment at a dose of 10 mg/kg was associated with significant increases in GPX1 and catalase protein expression in Barret’s metaplasia. Similarly, Abdellatif et al^39^ demonstrated the inhibitory effect of UDCA on DCA induced NF-κβ and its translocation, DCA induced Activator Protein-1 (AP-1) activation, induce upstream signaling proteins in esophageal cells.

A study by Goldman et al^40^ suggests that replacing hydrophobic bile acid with glycosodeoxycholic acid (GUDCA) is cytoprotective by reducing cell death, DNA damage, and oxidative stress, which are usually induced by bile and gastric acid.

In contrast to these protective effects of UDCA, it has been shown that among a cohort of 9 patients with known BE for 9 years who were pretreated with high doses of PPI and 600 mg of UDCA twice daily for 6 months, several outcomes were not altered (clinical, biochemical, and histological).^41^ Furthermore, Bozikas et al^41^ reported that GERD health-related quality of life did not significantly change in relation to the following outcomes: pH measurement, composition of bile salts, inflammatory markers (as demonstrated by low expression of COX-2), cellular proliferation by ki67 index, differentiation with absence of villin expression nor histological downgrading of BE dysplasia of cells. In a cohort of 29 patients who had been pretreated with PPI for 6 months, Banerjee et al^42^ confirmed the similar findings by demonstrating that UDCA at a dose of 13-15 mg/kg/day did not improve BE pathology grade, oxidative DNA damage, cellular proliferation, or apoptosis as demonstrated by Cleaved Caspase 3 (CC3). A subgroup of the cohort used Aspirin in addition to the intervention drugs that were noted to alter the concentrations of DCA and its glycine and taurine conjugates within the bile acid composition, however that did not alter the study outcomes.^42^

### B. Gastric disorders

The gastric lumen is constantly exposed to acid as a result of the release of HCl from the parietal cells and the reflux of duodenal contents containing pancreatic and biliary juices. Limited studies have demonstrated bile acid exposure-effect on the mucosa of the stomach under a few conditions, and thus the potential therapeutic benefits of UDCA.

**B1. Gastritis**. A cohort of 12 patients who underwent a Billroth II gastrectomy received UDCA at a dose of 1000 mg/day for 4 weeks while taking no other acid inhibitory medications (PPI, antacid) or cholestyramine. In addition to significant reductions in cholic acid (CA), DCA, and litholic acid (LA), significant improvements were observed in symptoms scores. However, no histological changes were noted with UDCA treatment.^43^

**B2. Helicobacter pylori infection**. In a group of outpatients with upper GI symptoms and documented uneradicated helicobacter pylori infection (n=40 patients) received UDCA monotherapy at a daily dose of 300 mg for 28 days without significant reductions in helicobacter pylori density, mononuclear cellular infiltration, or polymorphonuclear infiltration.^44^

**B3. Functional dyspepsia**. In a randomized placebo-controlled study, Kim et al^45^ examined the effects of this medication at a dose of 300 mg daily for 2 months in 24 patients with small intestinal bacterial overgrowth (SIBO). Compared to placebo, there was a statistically significant decrease in functional dyspepsia index as well as a decrease in methane and hydrogen producing SIBO patients. Furthermore, Aggio et al^46^ in 1986 evaluated symptom response in 26 patients using UDCA at 300 mg/d or placebo and demonstrated better symptom improvement with UDCA (55%) versus placebo (21%).

### C. Duodenal disorders

Limited studies evaluated the effects of UDCA on the duodenum for acid peptic disorders. These studies mostly reported on the role of UDCA in familial polyposis syndrome affecting the duodenum.

**C1. Familial adenomatous polyposis**. The effect of mucosal growth and dysplasia is evaluated in 4 studies.^47-50^ Ursodeoxycholic acid was used in post proctocolectomy FAP patients with duodenal adenomas who were treated at a dose of 10 mg/kg/day compared to placebo for 24 months, and then were evaluated endoscopically for regression of the duodenal polyps using the Spigelman severity score. At the conclusion of the study, 9 patients who were treated with UDCA versus 7 patients treated with placebo demonstrated no superiority benefit of UDCA.^50^ Comparatively, in a pilot study with 5 patients using high doses of UDCA of 25 mg/kg, it was noted that the expression of duodenal mucosal cyclooxygenase-2 (COX-2) was reduced by staining. Ursodeoxycholic acid cytotoxicity of bile acids had been significantly attenuated post intervention.^49^

Celecoxib is a cyclooxygenase-2 inhibitor with antioxidant properties that was evaluated in conjunction with UDCA to evaluate duodenal FAP. First, in 37 patients with documented FAP using endoscopy or APC gene documentation, Celecoxib combination therapy at a dose of 800 mg along with UDCA doses ranging from 1000 to 2000 mg daily is compared to celecoxib 800 mg daily. Moreover, placebo showed that the latter combination exerted reduction of duodenal polyp density, reduction of cellular proliferation (using Ki67), reduction of apoptosis (using cleaved cytokeratin 18), and reduction in COX-2 expression as a tumorigenic marker as compared to Celecoxib and UDCA. As a result, high doses of UDCA counteract Celecoxib’s effects.^47^ Second, researchers from the same group explored several markers of genes associated with normal mucosal tumorigenesis in FAP patients compared to controls without FAP. At the normal mucosa of FAP, mRNA levels of GSTA1 (a detoxification enzyme) and caspase-3 (an apoptotic marker) are significantly lower, indicating a reduced capacity to detoxify carcinogens and toxins. These genetic markers were not influenced by UDCA at a dose of 20-30 mg/kg and Celecoxib 800 mg daily compared to Celecoxib and placebo.^51^

### Dosage and Side effects

Oral doses of UDCA were stated in 9 studies.^38,41-46,47,49,51^ They were reported either as weight based or fixed doses. Weight based dosing ranged between 10 mg/kg/day 38 for chemoprevention of Barret’s esophagus indication up to 20-30 mg/kg/day for prevention of dysplastic changes in FAP.^51^ Fixed doses ranged between oral doses of 300 mg daily for indication of treatment of functional dyspepsia and SIBO 52 and non-organic dyspepsia 46 up to 1000 mg for the indication of treatment of Bile reflux gastritis.^43^ Intravenous UDCA was not reported as an indication of UGI disorders.

Four studies had detailed and reported the side effects of UDCA.^38,41,42,51^ The overall side effects profile (Table 1) is dominated by GI side effects (20 events, 50%).

**Table 1:** - Consolidated Standards of Reporting Trials 2010 checklist of information to include when reporting a randomized trial.

Topic	Item No.	Checklist item	Banerjee 2016	Parc 2011	Kim 2020	Vann 2013	Peng 2014	Bjorn 2013	Aggio 1986	Berkhout 2007
Title and abstract	1a	Identification as a randomized trial in the title	Missing	1	1	1	Missing	Missing	Missing	Missing
	1b	Structured summary of trial design, methods, results, and conclusions (for specific guidance see CONSORT for abstracts)	Missing	1	Missing	1	Missing	1	Missing	Missing
***Introduction***										
Background and objectives	2a	Scientific background and explanation of rationale	2	1,2	1,2	2	2	2	1	1
	2b	Specific objectives or hypotheses	3	2	2	2	2	2	1	1
Trial design	3a	Description of trial design (such as parallel, factorial) including allocation ratio	3	2	2	3	Missing	2	Missing	Missing
	3b	Important changes to methods after trial commencement (such as eligibility criteria), with reasons	Missing	Missing	Missing	Missing	Missing	Missing	Missing	Missing
Participants	4a	Eligibility criteria for participants	3	2	2	2,3	2	3	1,2	1
	4b	Settings and locations where the data were collected	3	2	2	2	Missing	3	Missing	Missing
Interventions	5	The interventions for each group with sufficient details to allow replication, including how and when they were actually administered	4	2	2,3	3	2,3	3	1	1
***Methods***										
Outcomes	6a	Completely defined pre-specified primary and secondary outcome measures, including how and when they were assessed	4,5	2	3,4	3,4	3,4	3	2	1
	6b	Any changes to trial outcomes after the trial commenced, with reasons	Missing	Missing	Missing	Missing	Missing	Missing	Missing	Missing
Sample size	7a	How sample size was determined	Missing	3	Missing	Missing	4	Missing	Missing	Missing
	7b	When applicable, explanation of any interim analyses and stopping guidelines	Missing	Missing	Missing	Missing	Missing	Missing	Missing	Missing
Randomization sequence generation	8a	Method used to generate the random allocation sequence	Missing	3	Missing	3	2	Missing	Missing	Missing
	8b	Type of randomization; details of any restriction (such as blocking and block size)	Missing	Missing	2	3	Missing	Missing	Missing	Missing
Allocation concealment mechanism	9	Mechanism used to implement the random allocation sequence (such as sequentially numbered containers), describing any steps taken to conceal the sequence until interventions were assigned	Missing	3	Missing	3	Missing	Missing	Missing	Missing
Implementation	10	Who generated the random allocation sequence, who enrolled participants, and who assigned participants to interventions	Missing	Missing	Missing	3	Missing	Missing	Missing	Missing
Blinding	11a	If done, who was blinded after assignment to interventions (for example, participants, care providers, those assessing outcomes) and how	Missing	3	Missing	3	Missing	2	1	Missing
	11b	If relevant, description of the similarity of interventions	Missing	3	Missing	3	Missing	3	1	Missing
Statistical methods	12a	Statistical methods used to compare groups for primary and secondary outcomes	5	3	4	4	4	3	2	Missing
	12b	Methods for additional analyses, such as subgroup analyses and adjusted analyses	Missing	3	Missing	Missing	Missing	Missing	Missing	Missing
***Results***										
Participant flow (a diagram is strongly recommended)	13a	For each group, the numbers of participants who were randomly assigned, received intended treatment, and were analyzed for the primary outcome	5	3,4	3	4,5	4,5,11	4	2	Missing
	13b	For each group, losses and exclusions after randomization, together with reasons	5	3,4	3	4,5	4,5,11	4	2	Missing
Recruitment	14a	Dates defining the periods of recruitment and follow-up	5	3	Missing	2	Missing	Missing	Missing	Missing
	14b	Why the trial ended or was stopped	Missing	3	5	4	Missing	Missing	Missing	Missing
Baseline data	15	A table showing baseline demographic and clinical characteristics for each group	11	4	4	6	Missing	4	Missing	Missing
Numbers analyzed	16	For each group, number of participants (denominator) included in each analysis and whether the analysis was by original assigned groups	5,6	3,4	4,5	4,5	Missing	4	2	2
Outcomes and estimation	17a	For each primary and secondary outcome, results for each group, and the estimated effect size and its precision (such as 95% confidence interval)	6,7	3,5	5	4,5,6	Missing	4	Missing	Missing
	17b	For binary outcomes, presentation of both absolute and relative effect sizes is recommended	Missing	3	5	Missing	Missing	Missing	Missing	Missing
Ancillary analyses	18	Results of any other analyses performed, including subgroup analyses and adjusted analyses, distinguishing pre-specified from exploratory	Missing	3	Missing	Missing	Missing	Missing	Missing	Missing
Harms	19	All important harms or unintended effects in each group (for specific guidance see CONSORT for harms)	Missing	3	Missing	6,8	Missing	Missing	Missing	Missing
***Discussion***										
Limitations	20	Trial limitations, addressing sources of potential bias, imprecision, and, if relevant, multiplicity of analyses	8	5,6	8,9	9	8	6,7	Missing	2
Generalizability	21	Generalizability (external validity, applicability) of the trial findings	Missing	Missing	Missing	Missing	Missing	Missing	Missing	Missing
Interpretation	22	Interpretation consistent with results, balancing benefits and harms, and considering other relevant evidence	7,8	5	8,9	7,8,9	7	6	3	2
***Other information***										
Registration	23	Registration number and name of trial registry	1	1,2	3	1,2	2	1	Missing	Missing
Protocol	24	Where the full trial protocol can be accessed, if available	Missing	1	Missing	Missing	Missing	2	Missing	Missing
Funding	25	Sources of funding and other support (such as supply of drugs), role of funders	9	6	9	10	8	6	Missing	Missing
		Overall CONSORT score (out of 37)	18	31	21	28	14	21	12	8

## Discussion

Our study explored a potential use of the secondary bile acid UDCA in the management of UGI disorders from a clinical point of view. Despite the reported low side effects profile of this medication, the review found a limited number of clinical studies. From a clinical, biochemical, and histopathological perspective, gastroesophageal reflux and its complications are not improved with the use of UDCA. However, it has been demonstrated that UDCA is beneficial when taken with PPIs in the setting of Barret’s esophagus, a known complication of GERD. UDCA is cytoprotective and has antioxidant properties when taken with PPIs. Despite these positive biological findings, there is no recommended international guideline that endorses its use as a chemopreventive agent. This is explained in part by the lack of a well performed longitudinal studies that address confounders and address the ongoing changes from several domains such as clinical, oncogenic and histopathological features. It is arguable that the genetic methods used to study the alteration of Barret cells would be better studied using more robust evaluation methods of the cellular and dysplastic growth than classically reported in the studies herein, this is probably achieved by studying chromosome instability, genome alteration and whole genome doubling or aneuploidy rather than using 8 hydroxy dG for evaluation of DNA oxidative damage, Ki67 for evaluation of cellular proliferation and CC3 for evaluation apoptosis.^52^

For gastric disorders, especially the studies addressing functional non organic dyspepsia reported symptomatic improvement with use of UDCA mainly for abdominal pain; however, nausea as a component of dyspepsia symptomatology was not examined leaving a gap in the literature. Regarding Helicobacter pylori infection, UDCA exerts no effect on colonization nor their related acute and chronic inflammatory reaction.^53^ There is no known long-term outcome observed for the symptomatic improvement observed with UDCA in esophageal and gastric disorders studied. Studies investigating duodenal disorders revealed only studies that examined FAP of the duodenum, while heterogeneous studies showed different doses and treatments alone or in combination with Celecoxib. Low doses (10-25 mg/kg/day) in a small number of cohorts showed regression of duodenal polyps’ density.^49,50^ Conversely, higher doses of UDCA when combined with Celecoxib exerted no plausible capability to detoxify carcinogens and toxins nor reduce polyp density or their growth parameters. When considering the rapid pharmacokinetics of UDCA with their early bioavailability within 40 minutes of intake and subsequently jejunal UDCA level that varies according to the ingested amount of UDCA that is predominantly fecally excreted, that translates into a rapidly processed drug effect, it would have been more useful to consider high UDCA dosages within the study protocols that evaluated chemopreventive properties effect on BE and FAP.^54^ These negative chemopreventive outcomes for the management of these 2 premalignant upper GI conditions stimulate further effort to utilize UDCA as a potential future chemopreventive agent. A previous systemic review, McQuaid et al^55^ concentrated on the effects of bile acids on pathobiology and oncogenesis in relation to Barret’s esophagus.

### Study limitation

This systematic review is the small number of human studies that investigated UDCA interventions, some of which were old publications. Due to a lack of unified accepted evaluation criteria for therapeutic effectiveness and oncogenic transformation of the premalignant conditions studied, there are limited data pertinent to its outcome on upper GI disorder management in a large cohort in order to make meaningful therapeutic recommendations.

In Conclusion, UDCA is a promising therapeutic agent to supplement the treatment armamentarium of functional dyspepsia when other causes are ruled out. With limited data and recommendations, its use could not be recommended as a chemopreventive agent to alter the oncogenic transformation of Barret’s esophagus and Familial adenomatous polyposis. Future studies are needed to address the chemopreventive properties of UDCA using more consistent and replicable biological tools.
